# Blood–brain barrier and brain structural changes in lung cancer patients with non-brain metastases

**DOI:** 10.3389/fonc.2022.1015011

**Published:** 2022-10-18

**Authors:** Da-Fu Zhang, Huan Ma, Guang-Jun Yang, Zhi-Ping Zhang, Yin-Fu He, Mao-Yang Feng, Bao-Ci Shan, Xiu-Feng Xu, Ying-Ying Ding, Yu-Qi Cheng

**Affiliations:** ^1^ Department of Psychiatry, the First Affiliated Hospital of Kunming Medical University, Kunming, China; ^2^ Department of Radiology, the Third Affiliated Hospital of Kunming Medical University, Yunnan Cancer Hospital, Yunnan Cancer Center, Kunming, China; ^3^ Department of Psychiatry, the Second Affiliated Hospital of Kunming Medical University, Kunming, China; ^4^ Laboratory of Nuclear Analysis Techniques, Institute of High Energy Physics, Chinese Academy of Sciences, Beijing, China; ^5^ Yunnan Clinical Research Center for Mental Disorders, The First Affiliated Hospital of Kunming Medical University, Kunming, China

**Keywords:** lung cancer, brain structure, blood-brain barrier, brain metastasis, dynamic contrast-enhanced MRI

## Abstract

**Purpose:**

To explore the relationship between blood-brain barrier (BBB) leakage and brain structure in non-brain metastasis lung cancer (LC) by magnetic resonance imaging (MRI) as well as to indicate the possibility of brain metastasis (BM) occurrence.

**Patients and methods:**

MRI were performed in 75 LC patients and 29 counterpart healthy peoples (HCs). We used the Patlak pharmacokinetic model to calculate the average leakage in each brain region according to the automated anatomical labeling (AAL) atlas. The thickness of the cortex and the volumes of subcortical structures were calculated using the FreeSurfer base on Destrieux atlas. We compared the thickness of the cerebral cortex, the volumes of subcortical structures, and the leakage rates of BBB, and evaluated the relationships between these parameters.

**Results:**

Compared with HCs, the leakage rates of seven brain regions were higher in patients with advanced LC (aLC). In contrast to patients with early LC (eLC), the cortical thickness of two regions was decreased in aLCs. The volumes of twelve regions were also reduced in aLCs. Brain regions with increased BBB penetration showed negative correlations with thinner cortices and reduced subcortical structure volumes (P<0.05, R=-0.2 to -0.50). BBB penetration was positively correlated with tumor size and with levels of the tumor marker CYFRA21-1 (P<0.05, R=0.2–0.70).

**Conclusion:**

We found an increase in BBB permeability in non-BM aLCs that corresponded to a thinner cortical thickness and smaller subcortical structure volumes. With progression in LC staging, BBB shows higher permeability and may be more likely to develop into BM.

## 1 Introduction

Brain metastasis (BM) are a serious public health problem on a global scale. It is estimated that approximately 20% of patients with cancer experience BM (1, 2), and that this is an important cause of cancer-associated mortality. Lung cancer (LC) is the most common cause of BM (3, 4). Approximately 25%-50% of LC patients have BM (5, 6). BMs may cause a range of focal neurological symptoms as well as cognitive impairment, thus greatly reducing patients’ quality of life (7). The average survival time of untreated patients with BM is 2-3 months (8, 9). Even with existing treatments (e.g., surgery, radiotherapy, chemotherapy, targeted therapy, immunotherapy), the median survival time of patients with BM is only approximately 5 months (10).

The pathogenesis of BM are complicated, and BBB dysfunction is considered one of its mediating mechanisms. LC cells reach the vascular system of the brain through blood circulation, attach to microvascular endothelial cells, infiltrate the parenchyma, induce angiogenesis, proliferate in response to growth factors, and finally cross the BBB to form intracerebral metastases (11).

The BBB is composed of endothelial cells, a basement membrane, an astrocytic foot, and pericytes (12, 13). Its integrity is essential for blocking the entry of toxic substances into the peripheral circulation as well as blocking most tumor cells (14). However, metastatic cells can cross the multiple cell layers that comprise the BBB, including through the proteolysis of adhesion molecules (such as JAM-B, junctional adhesion molecule B) (15), leukocyte mimicry (15), and through the action of a variety of cytokines (e.g., cyclooxygenase2 (COX2, also known as PTGS2), heparin-binding EGF-like growth factor (HB-EGF), ST6GALNAC5, PLEKHA5, placental growth factor (PLGF)) (16, 17).

The process of transferring cells across the BBB leads to the destruction of the BBB as well as to an increase in permeability. Therefore, effective measurement of BBB permeability may potentially have application and clinical utility in terms of predicting BM. Although BBB leakage plays an important role in BM, it is difficult to measure human BBB permeability. The ratio of cerebrospinal fluid to serum albumin is a common and well- developed method for evaluating the permeability of the BBB. However, it is invasive and its reliability is controversial as it is easily affected by cerebrospinal fluid flow (18).

Progress in neuroimaging technology to date has suggested the utility of a direct, quantitative, and detailed method for evaluating BBB functionality (19). More specifically, DCE-MRI can quantify the spillover of contrast medium to the brain parenchyma and measure lower-level BBB leakage/permeability (20, 21). DCE-MRI has been successfully applied to study diseases related to BBB dysfunction, such as multiple sclerosis (22), stroke (23), traumatic brain injury (20), and dementia (24). Research on tumors is mainly focused on evaluating tumor grade and patient prognoses, distinguishing changes after treatment (e.g., progression, tumor recurrence), and evaluating treatment efficacy and curative effects (25, 26).

Although the results of research to date are encouraging, the aforementioned methodology has rarely been used in evaluating BMs. Instead, this methodology has mainly been deployed in evaluating therapeutic efficacy with respect to BMs. However, to the best of our knowledge, there have been few studies on BBB microleakage prior to BM or on micro-metastasis.

In the current study, we hypothesized that BBB permeability may increase prior to BM or micro-metastasis and that BBB permeability is related to the primary LC stage. Specifically, we hypothesized that a higher LC stage would be associated with more significant BBB damage and a greater likelihood that this damage would be to develop into BM. DCE-MRI volume transfer constant (K*^trans^*) was used to detect changes in the BBB in order to predict the possibility of BM. Therefore, the purpose of this study was to quantify BBB permeability in LC patients without BM, and to explore the relationships between BBB permeability, brain structural changes, tumor staging, and tumor markers.

## 2 Materials and methods

### 2.1 Participants

This study was approved by the ethics committee of the third affiliated Hospital of Kunming Medical University (NO. SLKYLX202118). All participants provided their written informed consent prior to participating in this study. This work was conducted in accordance with the principles of the Declaration of Helsinki and its later amendments.

**Figure 1:**
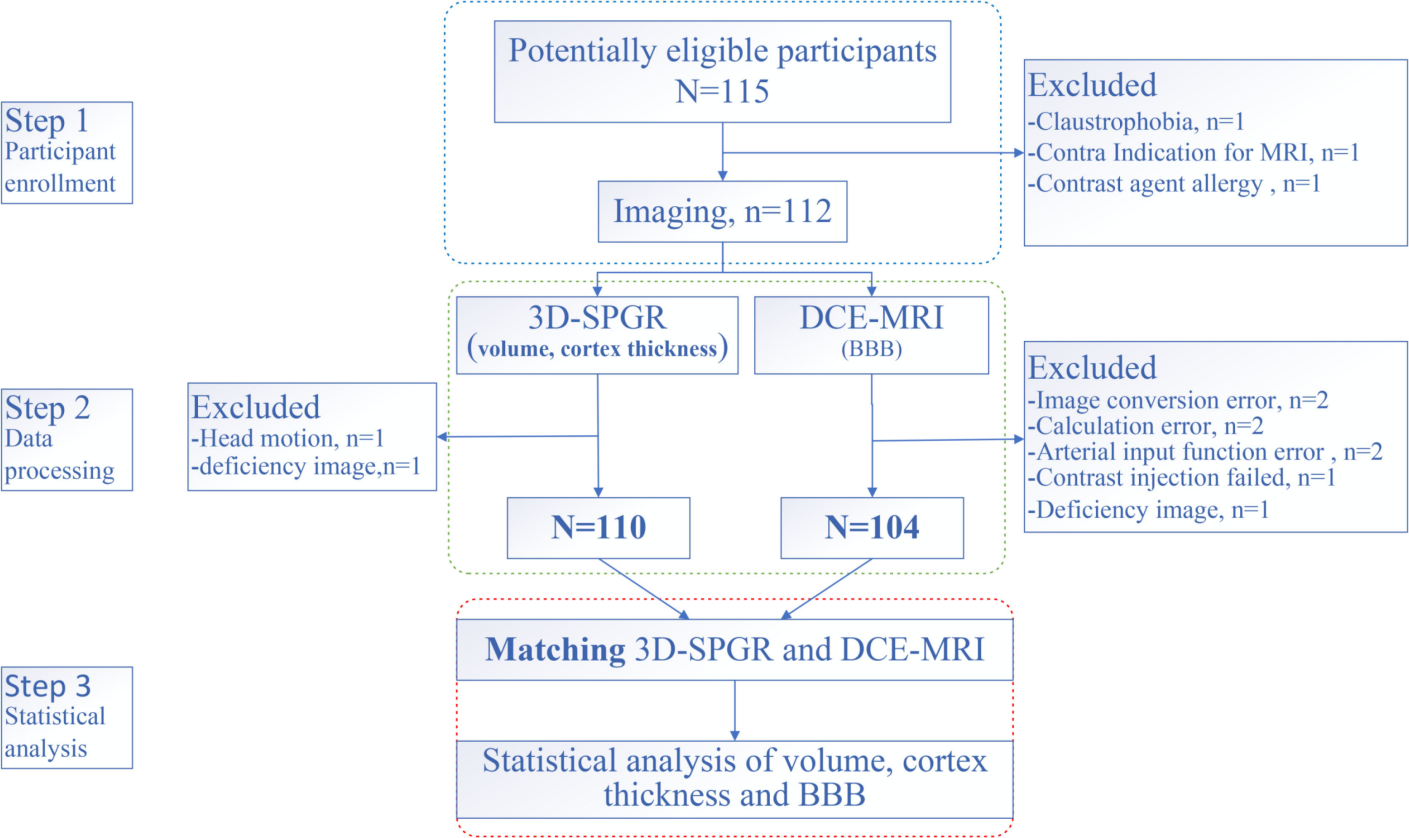
Study flowchart.

We conducted a cross-sectional study in LC patients. MRI was performed in untreated LC patients without a BM. We also enrolled age-and sex-matched HCs. Patients were enrolled into a group that had not received any treatment (e.g., surgery, radiotherapy, chemotherapy, immunotherapy) and had received a pathological diagnosis. Exclusion criteria were prophylactic craniocerebral irradiation, BM, stroke, craniocerebral trauma, epilepsy, Alzheimer’s disease, Parkinson’s disease, other acute mental or neurological diseases, a history of major medical diseases (e.g., anemia, severe heart disease, thyroid dysfunction, abnormal liver or kidney function), and severe vision or hearing loss. According to the TNM (tumor, node, metastasis) staging criteria (27), early-stage patients were categorized into stage I LC, and those in more advanced stages were categorized into stage II–IV LC(Supplementary material for detail). The study flowchart is shown in **Figure 1**.

### 2.2 Imaging

#### 2.2.1 MRI acquisition

Images were acquired using a 3T MRI scanner (Discovery MR750, GE Healthcare, Waukesha, Wisconsin, USA) with a 21-channel MR Instruments head coil. Tight but comfortable foam pads was used to minimize head movement and earplugs and headphones were used to reduce scanner noise. Participants were instructed to lie down with their eyes closed, to stay awake, and not to think of anything special. For each participant, routine MRI sequences, including T2 and T1-weighted imaging and T2 fluid attenuated inversion recovery (FLAIR) imaging, was performed to ensure that there were no visible brain lesions or BMs.

The sequences for BBB assessment included the following steps: (1) a T1- weighted three-dimensional (3D) axial anatomical scan (BRAVO, TR=8.5ms, TE=3.2ms, field of view (FOV) 25.6×25.6, acquisition matrix 256×256, voxel size 1×1×1 mm, bandwidth 31.25kHz, FA 12°; (2) a T1- weighted 3D axial sequence with variable flip angles (3D-SPGR, TR 5.9ms, TE 2.0ms, flip angle 5° and 14°, FOV 24×20.4, acquisition matrix 256×180, slice thickness 4.0 mm, interval 0, bandwidth 62.5kHz); and (3) a T1- weighted 3D axial dynamic scan (LAVA, TR 5.9ms, TE 2.0ms, flip angle 14°, FOV 24×20.4, acquisition matrix 256×180, slice thickness 4.0 mm, interval 0, bandwidth 62.5 kHz) acquired within 650 s after intravenous injection of the magnetic contrast gadolinium (0.05 mmol/kg, flow rate 3.0 mL/s).

#### 2.2.2 Analysis of the cerebral cortex and subcortical structures

High-resolution T1-weighted anatomical images were processed using the FreeSurfer 7.2 software package (https://surfer.nmr.mgh.harvard.edu/). Processing included: 1) motion correction and averaging of multiple volumetric T1-weighted images; 2) removal of non-brain tissue using a hybrid watershed/surface deformation procedure; 3) automated Talairach transformation; 4) segmentation of the subcortical white matter and deep gray matter volumetric structures (including the hippocampus, amygdala, caudate, putamen, ventricles); 5) intensity normalization; 6) tessellation of the gray matter white matter boundary with automated topology correction; and 7) surface deformation following intensity gradients to optimally place the gray/white and gray/cerebrospinal fluid borders at the location where the greatest shift in intensity defines the transition to the other tissue class. Cortical thickness and subcortical structure volume were calculated using the software template [i.e., the Destrieux atlas (28)]. We analyzed the cortical thicknesses of 74 structures as well as 16 subcortical volumes.

#### 2.2.3 BBB data processing

The contrast medium leakage caused by BBB leakage leads to an increase in the T1-weighted signal in the affected tissue, thus enabling the contrast medium leakage to be calculated. To achieve this, we used SPM12 to register and normalize T1-weighted images acquired continuously after contrast injection to the MNI (Montreal Neurological Institute) coordinates (University College London, www.fil.ion.ucl.ac.uk/spm).

Previous studies have shown that the Patlak model is more accurate than other models in diseases presenting with slight BBB damage (29–31). Using DCE-MRI to measure the subtle leakage of the BBB has moderate to excellent repeatability (32). Therefore, in this study, the Patlak model was used to calculate K*^trans^*. All dynamic images were registered to the same reference image, with an average flip angle of 14° to correct for head displacement. The Patlak method uses a two-compartment model, in which it is assumed that there is no reflux and infinite flow. Therefore, the leakage rate is similar to the product of vascular permeability (P) and the surface area (S) per unit tissue mass.

**Figure 2:**
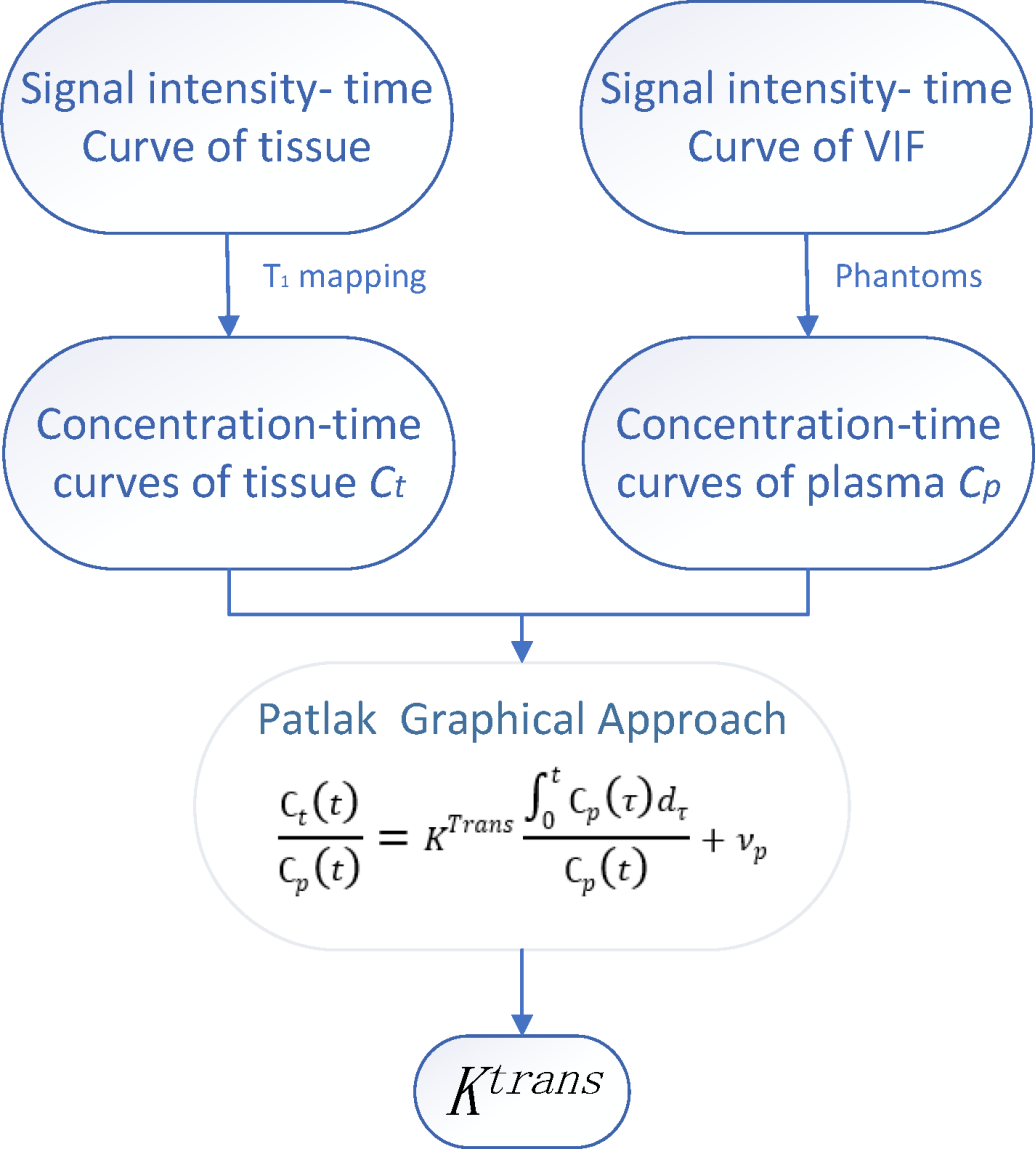
The time-signal intensity curve of tissue and vascular input function (VIF) was converted into time-concentration curve. The leakage rate (K^trans^) and plasma volume (Vp) were calculated by Patlak graphic method.

For the Patlak graphic method, the two main factors affecting the accurate measurement of BBB permeability are the estimation of the blood concentration curve based on T1 signal intensity and the determination of the VIF (variance inflation factor) (33) (**Figure 2**). A common method of T1 mapping is to change the flip angle (34). VIF measurements also play a key role in estimating kinetic parameters. VIF was calculated by selecting the region of interest (ROI) of the superior sagittal sinus (35). The sagittal sinus has a sufficiently large cross-section such that the VIF can be easily extracted from the superior sagittal sinus and is not affected by partial volume and inflow artifacts (35). Thereafter, *K^trans^* (min^-1^) was calculated using MATLAB software (MathWorks, Natick, MA, USA), implementing the Patlak model. The *K^trans^* value was used to reflect BBB leakage.

*K^trans^* is calculated as a voxel. The whole brain was divided into 116 brain regions based on the AAL atlas (36). Each brain region was considered as a ROI with respect to extracting the average *K^trans^* value for statistical analysis.

## 3 Statistical analysis

SPSS (version 19.0; SPSS Inc., Chicago, IL, USA) and R statistical software (version 4.1.2, The R Project for Statistical Computing, Vienna, Austria; https://www.r-project.org/) were used for statistical analyses. Continuous variables with normal distributions are described as mean ± standard deviation. We evaluated classification variable usage (%) or the constituent ratio (%). If the data were normally distributed and the variance was uniform, an analysis of variance (ANOVA) test was implemented and LSD Test was used for *post hoc* multiple comparisons. Otherwise, the Kruskal-Wallis (KW) test was performed. Differences were considered statistically significant at a two-sided P-value of <0.05. The results were visualized using R statistical software or GraphPad Prism visualization software (San Diego, CA, USA). Multiple comparisons were performed with Bonferroni correction.

## 4 Results

### 4.1 Demographic and clinical characteristics

A total of 75 LC patients (39 eLCs and 36 aLCs) and 29 HCs (selected through online advertising) were enrolled from August 2021 to March 2022 at the Department of Thoracic Surgery of the Third Affiliated Hospital of Kunming Medical University (Kunming, China). Participants were age and sex matched. Eleven subjects were excluded due to excessive head movement, allergies to the contrast medium, or miscalculation during scanning. The number of participants assessed for eligibility and the reasons for exclusion is shown in **Figure 1**.

A summary of detailed demographic data, histological diagnoses, and tumor staging is shown in **Table 1**. There were no statistically significant differences in sex, age, smoking, or KPS (Karnofsky Performance Scale) scores between the LC patients and the control group (P>0.05). The tumor diameter in the eLC group was smaller than that in the aLC group (P<0.001). The levels of the tumor markers CEA (carcinoembryonic antigen), NSE (neuron-specific enolase), CYFRA21-1 (cytokeratin 19 fragment), and SCC (squamous cell carcinoma antigen) were higher in the aLC group than those in the eLC group (P<0.05).

**Table 1:** Demographic and clinical features of patients with lung cancer.

	Controls (n = 29)	Early lung cancer (n = 39)	Advanced lung cancer (n = 36)	χ2/F/μ	pvalue
**gender**					
male/female	15/14	15/24	24/12	5.986	0.05^a^
**Age(mean ± SD), year**	51.07 ± 9.67	55.59 ± 8.23	56.06 ± 8.99	3.059	0.051^b^
**Smoking(%)**	10/29	9/30	16/20	3.84	0.147^a^
**Tumor diameter (cm) (mean ± SD)**		1.62 ± 0.94	4.93 ± 2.35	-8.103	0.000^*c^
**KPS score(mean ± SD)**	95.00 ± 7.77	96.15 ± 7.11	94.44 ± 6.95	0.568	0.568^b^
**Clinical stage**					NA^d^
I		39	0		
II		0	4		
III		0	20		
IV		0	12		
**Pathological type**				χ2	
Squamous cell carcinoma		7	13	7.396	0.092^a^
Adenocarcinoma		29	18		
Small Cell Lung Cancer		3	5		
**Tumor markers (25%,50%,75%)**				μ	
CEA		1.53, 2.25, 3.57	1.87,4.38, 10.33	446.50	0.007^*e^
NSE		9.60, 11.80, 13.40	11.75, 13.70,21.96	397.50	0.001^*e^
CYFRA21-1		1,40, 2.00,2.60	2.625,4.500,7.025	223.00	0.000^*e^
SCC		0.70,0.80,1.00	0.72,1.10,1.47	493.00	0.026^*e^

Data are expressed as Mean ± SD, n (%) or InterQuartile Range(P_25_,P_50_,P_75_). ^a^The P values are obtained by using χ2 test. ^b^The P values are obtained by using one-way ANOVA. ^c^The P values are obtained by using two sample t-test. ^d^There is no statistical analysis. ^e^The P values are obtained by Kruskal-Wallis test. ^*^P<0.05 is considered significant. CEA, carcinoembryonic antigen; NSE, neuron-specific enolase; CYFRA21-1, cytokeratins21-1; SCC, squamous cell carcinoma antigen.

### 4.2 BBB leakage in patients with LC

**Figure 3:**
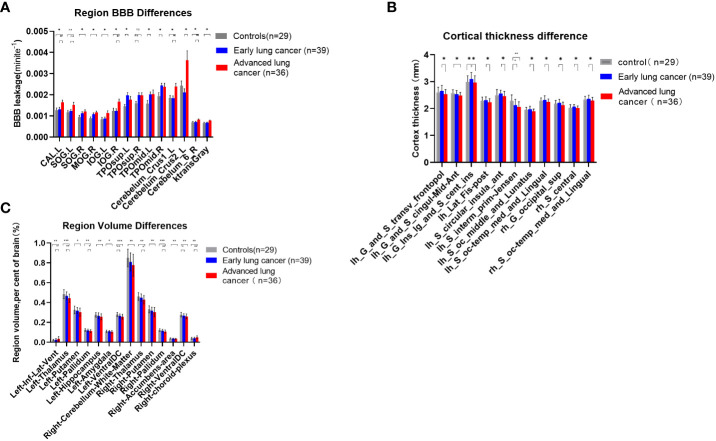
**(A)** Comparisons of BBB leakage between patients with LC at different stages and HCs. Quantify the leakage rate of BBB in different brain regions, and there were significant differences in 8 brain regions between patients with aLC and HCs (p < 0.05), which revealed the existence of BBB leakage in patients with aLC. There was no statistical difference between SOG.R and TPOsup.R after correction. **(B)** Comparisons of cerebral cortex thickness between patients with LC at different stages and HC. There was significant difference between patients with aLC and patients with eLC (p < 0.05). The thickness of 9 cerebral cortices decreased in patients with aLC. There was no statistical difference between the cerebral cortex of patients with LC and HC, but the cortex of patients with eLC showed an increasing trend and a decreasing trend in aLC. **(C)** Comparisons of subcortical structure volume between patients with LC at different stages and HC. There was significant difference between HC of patients with aLC (p < 0.05). In patients with aLC, the volume of 13 subcortical structures decreased, while the volume of 1 structure increased. The volume of bilateral VentralDC showed volume reduction in patients with eLC. The volume of 6 subcortical structures in patients with aLC was smaller than that in patients with eLC, while the volume of one structure increased in patients with aLC. *P<0.05, **P<0.01, ***P<0.001; ns, no statistical difference.

Compared with the control group, the K*^trans^* levels of seven brain regions and the whole cerebral gray matter in aLC group were higher than those in HC group (P<0.05) (i.e., the *left calcarine fissure and surrounding cortex (CAL.L), right superior occipital gyrus (SOG.R), right middle occipital gyrus (MOG.R), left inferior occipital gyrus (IOG.L), right inferior occipital gyrus (IOG.R), left Cerebelum Crus1, right Cerebelum 6*). The permeability of *left Temporal pole: superior temporal gyrus (TPOsup.L), right temporal pole: middle temporal gyrus (TPOmid.R), and TPOmid.L* was increased in eLC group (P<0.05). The permeability of *CAL.L, IOG.R, left Cerebellum Crus1, left Cerebellum Crus2 and right Cerebellum 6* was increased with the LC staging progression. There were no statistically significant differences in BBB permeability in the other brain regions (**Figures 3A**, **S1–S16**).

### 4.3 Changes in cerebral cortex thickness and volume

**Figure 4:**
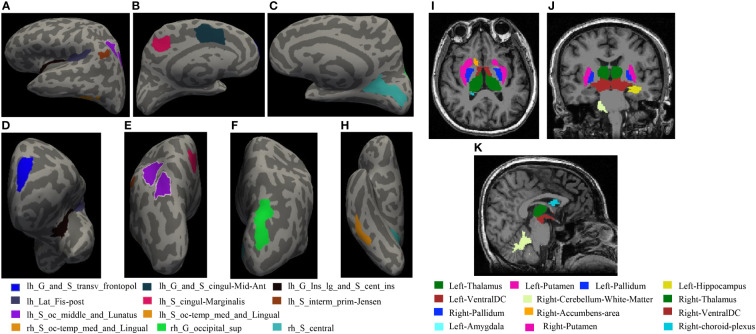
**(A–H)** shows statistical differences in the locations of cortical thickness: **(A)** Left hemisphere left side view; **(B)** Left hemisphere right side view; **(C)** Right hemisphere left side view; **(D)** Left hemisphere front view; **(E)** Left hemisphere rear view; **(F)** Right hemisphere rear view; H Right hemisphere bottom view. **(I–K)** The locations of subcortical structures with statistical differences: **(I)**: coronal, **(J)**: axial, **(K)**: sagittal.

A comparison of 74 cortical thicknesses between eLCs, aLCs and HCs showed that cortical thickness in nine brain regions in aLC group was smaller than that of eLC group (i.e., *left Transverse frontopolar gyri and sulci*, *left Long insular gyrus and central sulcus of the insula*, *left Posterior ramus of the lateral sulcus*, *left. Anterior segment of the circular sulcus of the insula*, *left. Middle occipital sulcus and lunatus sulcus*, *left. Medial occipito-temporal sulcus and lingual sulcus*, *right Superior occipital gyrus*, *right Central sulcus and right Medial occipito-temporal sulcus and lingual sulcus*)(P<0.05). The cortical thickness of the *left aMCC* and *the left sulcus intermedius primus (Jensen)* in aLC group was statistically significantly lower than in HC group. The cortical thickness of *the left long insular gyrus and the central sulcus of the insula* increased in eLC group (P<0.05). The locations are shown in **Figures 4A–H**). There were no statistical differences with respect to the other cortical thicknesses. However, we found an increasing trend in cortical thickness, as well as a decreasing trend in the LC group (**Figure 3B**, **Supplementary Material Tables S17–S19**).

Compared with HCs, the volume of 12 brain regions in aLC group decreased (i.e., *bilateral thalamus, putamen, pallidum, ventral diencephalon, left hippocampus, amygdala and right cerebellum white matter, nucleus accumbens)*, whereas the volume of the *left temporal horn of lateral ventricle* increased (P<0.05). The specific locations are shown in **Figures 4I–K**. In eLC group, the volume decreased preferentially in the *bilateral Ventral Diencephalon* (P<0.05), but there were no statistically significant differences with respect to other volume changes. The volumes of *the bilateral thalamus, right pallidum, and right choroid plexus* in aLC group were smaller than those in eLC group (**Figure 3C**; **Supplementary Material Tables S20–S22**; **Figures S1–S4**).

### 4.4 The correlations between the tumor diameter, serum marker, cortical thickness, volume and BBB leakage

**Figure 5:**
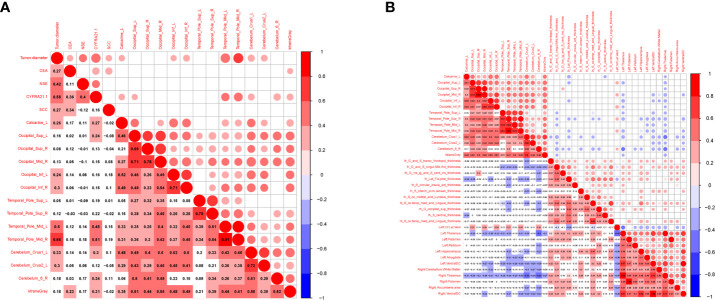
Correlation between tumor markers, differential cerebral cortical thickness, subcortical volume and BBB leakage in patients with LC. Red represents positive correlation, blue represents negative correlation, the darker the color, the higher the correlation. **(A)** Showed the correlation between tumor markers, tumor diameter and BBB leakage in LCs (n=75). **(B)** Showed the correlation between cerebral cortical thickness, subcortical volume and BBB leakage in LCs and HCs (n=104).

To examine whether the maximum diameter of the tumor, serum marker levels (CEA, NSE, CYFRA21-1, SCC), cortical thickness, and volume were related to BBB leakage, we analyzed the correlations between them. We found that the maximum diameter of the tumor was positively correlated with K*^trans^* (*CAL.L, IOG.L, IOG.R, TPOmid.L, left Cerebellum Crus2*). CEA was positively correlated with K*^trans^* of the cerebral gray matter, and CYFRA21-1 was positively correlated with *CAL.L, TPOmid.L, left cerebellum crus1, right cerebellum 6, and K^trans^ Gray* (P<0.05, R=0.25–0.51) (**Figure 5A** and **Supplementary Material Table S23**). Increased K*^trans^* brain area was negatively correlated with the average cortical thickness of *the left aMCC*, *the left long insular gyrus, the central sulcus of the insula, the posterior ramus of the lateral sulcus, the left sulcus intermedius primus, the left medial occipitotemporal sulcus, the lingual sulcus, the right superior occipital gyrus, and the volumes of the left thalamus, the left pallidum, the left hippocampus, the left ventral diencephalon, the right cerebellum (white matter), the right thalamus, the right accumbens, and the right ventral diencephalon.* Increased K*^trans^* brain area was also positively correlated with *the left temporal horn of lateral ventricle volume* (P<0.05, |R|=0.2-0.50) (**Figure 5B** and **Supplementary Material Table S24**).

## 5 Discussion

To the best of our knowledge, this is the first study to perform a quantitative analysis of BBB leakage in LCs without a BM. It was found that BBB leakage increased in patients with aLC and this was related to brain structure. Patients with aLC showed a higher level of BBB leakage in some brain regions. The BBB permeability was associated with a decrease in cerebral cortex thickness and volume. These findings present a key step in establishing the role of BBB dysfunction in the pathogenesis of BM in LC and highlight that the BBB may be a potential diagnostic and therapeutic target. BM can cause severe, uncontrollable symptoms and reduce quality of life, including by inducing paralysis, elevated intracranial pressure, and/or seizures. The incidence of BM has shown an upward trend in the past decade, but there has been little progress in treatment, and therapeutic effects have not been of sufficient quality (37). Therefore, it is particularly important to identify LC patients who are more prone to BM. Intensive treatment should be performed in these patients.

The occurrence of BM involves a series of interrelated steps, starting with the invasion of local cancer cells that progresses into the intravascular and/or circulatory and/or lymphatic system. Owing to the lack of a lymphatic system within the central nervous system, the only way for cancer cells to reach the brain is through blood circulation. The resulting circulating tumor cells (CTCs) may enter the microcirculation of the brain and adapt to the microenvironment of the brain tissue, thus resulting in the formation of micro-metastases that eventually form visible tumors through metastatic colonization (38, 39). However, metastatic cells that invade the parenchyma of the central nervous system must pass through the BBB. Cerebral vascular endothelial cells change in this process (40). These changes include damage to the tight junction structure and an increased perivascular space (41). In addition, windows corresponding to the surrounding vascular system can be found in these vessels, and the number and activity of pinocytic vacuoles have been shown to be increased (42). Therefore, these blood vessels may reflect the blood vessels of the tumor tissue rather than those of the endothelial cells of the central nervous system. Owing to these structural changes, the leakage of the BBB was found to increase in this study, and plasma was found to infiltrate into the extracellular space (43).

DCE-MRI can be used to evaluate the leakage of extracellular space in each voxel using pharmacokinetics parameters (K*^trans^*) as well as to detect BBB leakage (i.e., reflecting BBB destruction). K*^trans^* is defined as the volume transfer constant between the plasma and the extracellular space and is often used as a synonym for permeability. This parameter has been confirmed to be increased in patients with BMs (44), multiple sclerosis (22), stroke (23), traumatic brain injury (20), and dementia (24). Although DCE-MRI has been widely used in Neuro-oncology imaging, it is uncommon to measure relatively complete BBB leakage. Therefore, the level of leakage of BBB that we need to measure in order to establish prognoses may be small. Thus, the Patlak model was used to calculate K*^trans^* in the current study, as this model is more accurate in measuring low level leakage (29, 31).

Our results confirm that BBB leakage is increased in patients with LC, especially in aLC. It is suggested that, with the progression of LC, the integrity of the BBB may be destroyed in a wider range of brain regions and that permeability may increase. These changes indicate changes in cerebral vascular endothelial cells in these brain regions (26), damage to tight junction structures, and an increase in the perivascular space (27). This in turn indicates that the potential occurrence of microtransfer or a transition from the BBB to the blood-tumor barrier (BTB), because the BTB is generally considered to be more prone to leaking than the BBB (45). Therefore, BM may develop if there is no further clinical intervention. Early detection of increased BBB permeability as well as strengthening clinical interventions is of great significance in preventing the occurrence of BM.

We observed an interesting phenomenon in the current study. Compared with HCs, the cortical thickness of patients with eLC showed an increasing trend, although this difference was not statistically significant. Compared with patients with eLC, the cortical thickness of patients with aLC showed a downward trend, with statistically significant differences in the frontal transverse pole, the cingulate gyrus, the insular, the temporal pole, and the occipital pole cortices. At present, these findings have not been reported in the literature. Hence, our findings may represent a new discovery. However, these findings require more research support, including confirmed in studies with a larger sample size.

Our study further found that patients with aLC showed a larger tumor size, higher staging, and a higher incidence of BM compared with those with eLC (46, 47). With an increase in tumor diameter, BBB permeability was found to increase in the left peri-talar cortex, the bilateral suboccipital gyrus, the middle temporal pole, and the inferior cerebellum, probably because it is easier for tumor cells to invade the vascular or lymphatic system given a larger tumor volume (with CTCs entering the cerebral microcirculation). This may affect BBB integrity in the above-mentioned brain regions. CEA, CYFRA21-1, NSE, and SCC are biomarkers related to the LC and are suitable for LC screening and recurrence monitoring. In this study, the tumor markers CEA, NSE, CYFRA21-1, and SCC were high in advanced LC. BBB permeability of the cerebral gray matter increased with an increase in CEA and CYFRA21-1 levels. The BBB permeability of the left talar fissure, the left superior occipital gyrus, the bilateral middle temporal gyrus, and the superior cerebellum also increased with an increase in CYFRA21-1, indicating that the observed increase in serum tumor markers was correlated with BBB destruction. Therefore, the relationship between elevated tumor marker levels and BBB integrity may reflect tumor heterogeneity and may be a risk factor for BM (48, 49).

In contrast, in patients with aLC, cortical thickness and subcortical volume were smaller with increased BBB permeability. This may be because the development of LC is a long process, and that tumor tissue directly or indirectly affects the BBB by secreting cytokines or forming CTCs, which leads to changes in local microcirculation structure and hemodynamics, ultimately leading to a reduction in cortical structure and subcortical volume, an increase in space within this area, easier adhesion and retention of CTCs, and increased local invasion and micro-metastasis. The continuous expansion of tumor lesions causes local and distal changes, which directly damage the activity of neurons and vascular function (50), thus further aggravating leakage of the BBB and reducing the volume of the corresponding brain structure. However, the specific mechanisms underlying these changes are not clear. Therefore, BBB imaging may have the potential to identify biomarkers for BM risk in LC patients. Clinical adjuvant therapy should be strengthened for saboteurs of the BBB, including better chemotherapy for CTCs and prophylactic whole-brain radiotherapy for brain micro-metastasis, in order to achieve accurate individual treatment.

The advantages of this study include a lack of clear recruitment bias and a short time interval between clinical evaluation and neuroimaging regimens (usually after clinical evaluation). Moreover, MRI examination was completed within one week. However, the enrolled sample size of our cohort was small and our imaging scheme required a gadolinium-based contrast agent, which may potentially damage renal function and limit the wide applicability of this methodology.

## 6 Conclusion

In conclusion, this study provides convincing evidence of BBB leakage and its relationship with brain structure in patients with LC at different stages in patients without a BM. The accurate measurement of BBB leakage has the potential to be established as an effective biomarker for predicting BM.

## Data availability statement

The original contributions presented in the study are included in the article/**Supplementary Material**. Further inquiries can be directed to the corresponding authors.

## Ethics statement

The studies involving human participants were reviewed and approved by the ethics committee of the third affiliated Hospital of Kunming Medical University. The patients/participants provided their written informed consent to participate in this study.

## Author contributions

D-FZ: Data curation, Methodology, Software, Writing- Original draft preparation. Y-QC: Conceptualization, Writing- Reviewing and Editing. G-JY: Visualization, Investigation. Y-YD: Supervision. HM, X-FX: Writing- Reviewing and Editing. B-CS, M-YF: Software. Z-PZ, Y-FH: Data curation. All authors contributed to the article and approved the submitted version.

## Funding

This work was supported by National Natural Science Foundation of China (82060259, 81760296, 82001986); Yunnan Province High-Level Health Technical Talents (leading talents) (L-2019004, L-2019011); Yunnan Province Special Project for Famous Medical Talents of the “Ten Thousand Talents Program” (YNWRMY- 2018-040, YNWR-MY-2018-041); Yunnan digitalization, development and application of biotic resource (202002AA100007); The Outstanding Youth Science Foundation of Yunnan Basic Research Project (202101AW070001). Innovation Team of Kunming Medical University (CXTD202110). The Applied Basic Research Projects of Yunnan Province (2019FE001-083).

## Acknowledgments

This study is a joint effort of many investigators and staff members, and their contribution is gratefully acknowledged. We especially thank all patients who participated in this study. We thank Elsevier Webshop - Author Services for editorial and language assistance.

## Conflict of interest

The authors declare that the research was conducted in the absence of any commercial or financial relationships that could be construed as a potential conflict of interest.

## Publisher’s note

All claims expressed in this article are solely those of the authors and do not necessarily represent those of their affiliated organizations, or those of the publisher, the editors and the reviewers. Any product that may be evaluated in this article, or claim that may be made by its manufacturer, is not guaranteed or endorsed by the publisher.
