# Supplementary material for: Blood–brain barrier and brain structural changes in lung cancer patients with non-brain metastases
Source: Front Oncol. 2022 Oct 18;12:1015011. doi: 10.3389/fonc.2022.1015011 (PMC9623018; doi:10.3389/fonc.2022.1015011)
Supplement: Supplementary file 1 [file DataSheet_1.docx]

Supplementary Materials

TNM Staging

Stage Ⅰ, T_1a-2a_N_0_M_0_, longest diameter≤4cm, was defined as early lung cancer, no lymph node metastasis was found, so the prognosis was good. Adjuvant chemotherapy is not recommended for patients with stage I lung cancer after operation.

Stage Ⅱ-Ⅳ, T_2b-4_N_0-1_M_0-1_, Adjuvant chemotherapy is routinely recommended for patients in stage Ⅱ-Ⅳ.

The tumor size is a measure of the overall size of the tumor.

Table S1 Comparison of BBB leakage between patients with LCs and HCs

|  | HCs(N=29) | LCs(N=75) | U | P |
| --- | --- | --- | --- | --- |
| Lingual_L | 5.655E-04±2.810E-04 | 6.798E-04±2.730E-04 | 1360 | 0.048 |
| Occipital_Sup_R | 9.468E-04±4.000E-04 | 1.161E-03±5.080E-04 | 1415 | 0.018 |
| Occipital_Mid_L | 6.351E-04±3.350E-04 | 7.783E-04±3.220E-04 | 1380 | 0.034 |
| Occipital_Mid_R | 8.891E-04±3.660E-04 | 1.121E-03±3.790E-04 | 1467 | 0.006 |
| Angular_R | 1.021E-03±3.660E-04 | 1.268E-03±5.340E-04 | 1403 | 0.022 |
| Temporal_Pole_Sup_L | 1.451E-03±4.620E-04 | 1.875E-03±8.170E-04 | 1464 | 0.006 |
| Temporal_Pole_Sup_R | 1.594E-03±5.030E-04 | 1.983E-03±6.830E-04 | 1436 | 0.012 |
| Temporal_Pole_Mid_L | 1.579E-03±7.940E-04 | 2.026E-03±9.610E-04 | 1432 | 0.013 |
| Temporal_Pole_Mid_R | 1.938E-03±8.610E-04 | 2.406E-03±8.430E-04 | 1461 | 0.007 |
| ktransGray | 6.601E-04±2.020E-04 | 7.314E-04±1.620E-04 | 1371 | 0.040 |

Data are expressed as Mean±SD. The P values are obtained by using Independent-Samples Mann-Whitney U Test.

Comparison of BBB leakage between and HCs. The BBB leakage increased in 10 brain regions between LCs and HCs (P＜0.05).

*Occipital_Sup=* *Superior occipital gyrus; Occipital_Mid=* *Middle occipital gyrus; Temporal_Pole_Sup= Temporal pole: superior temporal gyrus; Temporal_Pole_Mid= Temporal pole: middle temporal gyrus; ktransGray= K^trans^ of gray.*

Table S2 Comparison of BBB leakage between patients with lung cancer at different stages and HCs.

|  | HCs(N=29) | eLCs(N=39) | aLCs(N=36) | H | P |
| --- | --- | --- | --- | --- | --- |
| CAL.L | 1.283E-03±5.090E-04 | 1.320E-03±5.120E-04 | 1.650E-03±6.100E-04 | 9.124 | 0.010 |
| SOG.L | 1.161E-03±5.640E-04 | 1.226E-03±5.320E-04 | 1.523E-03±7.360E-04 | 6.604 | 0.037 |
| SOG.R | 9.468E-04±4.010E-04 | 1.119E-03±5.140E-04 | 1.208E-03±5.030E-04 | 6.918 | 0.031 |
| MOG.R | 8.891E-04±3.660E-04 | 1.078E-03±4.020E-04 | 1.168E-03±3.520E-04 | 9.086 | 0.011 |
| IOG.L | 8.327E-04±5.410E-04 | 8.765E-04±4.410E-04 | 1.142E-03±5.790E-04 | 6.716 | 0.035 |
| IOG.R | 1.234E-03±7.140E-04 | 1.232E-03±6.680E-04 | 1.676E-03±7.340E-04 | 9.455 | 0.009 |
| TPOsup.L | 1.451E-03±4.620E-04 | 1.975E-03±7.640E-04 | 1.768E-03±8.680E-04 | 8.627 | 0.013 |
| TPOsup.R | 1.594E-03±5.030E-04 | 1.992E-03±6.550E-04 | 1.972E-03±7.210E-04 | 6.385 | 0.041 |
| TPOmid.L | 1.579E-03±7.940E-04 | 2.020E-03±7.270E-04 | 2.032E-03±1.174E-03 | 6.561 | 0.038 |
| TPOmid.R | 1.938E-03±8.610E-04 | 2.437E-03±6.600E-04 | 2.373E-03±1.014E-03 | 8.057 | 0.018 |
| Cerebelum_Crus1_L | 1.856E-03±6.300E-04 | 1.839E-03±6.810E-04 | 2.384E-03±9.040E-04 | 9.882 | 0.007 |
| Cerebelum_Crus2_L | 2.428E-03±1.211E-03 | 2.115E-03±1.046E-03 | 3.635E-03±2.650E-03 | 6.395 | 0.041 |
| Cerebelum_6_R | 7.005E-04±2.050E-04 | 7.119E-04±2.390E-04 | 8.212E-04±2.190E-04 | 8.193 | 0.017 |
| ktransGray | 6.601E-04±2.020E-04 | 6.875E-04±1.530E-04 | 7.788E-04±1.600E-04 | 9.610 | 0.008 |

Data are expressed as Mean±SD. The P values are obtained by using Kruskal-Wallis Test.

Comparison of BBB leakage between patients with lung cancer at different stages and HCs. There were significant differences in the ^Ktrans^ levels of 13 brain regions and the whole cerebral gray matter.

*CAL.L =* *Calcarine fissure and surrounding cortex，SOG.L=* *left Superior occipital gyrus; SOG.R=right Superior occipital gyrus，MOG.R=right* *Middle occipital gyrus; IOG.L=* *left Inferior occipital gyrus，IOG.R=right Inferior occipital gyrus; TPOsup.L=* *left Temporal pole: superior temporal gyrus，TPOsup.R=right Temporal pole: superior temporal gyrus，TPOmid.L=* left *Temporal pole: middle temporal gyrus，TPOmid.R=* right *Temporal pole: middle temporal gyrus，*

Table S3-16. Multiple comparisons of group. Asymptotic significances (2-sided tests) are displayed. The significance level is 0.05.

^a^. Significance values have been adjusted by the Bonferroni correction for multiple tests. 1= HCs; 2= eLCs; 3= aLCs.

A negative value means that the rank mean of the former group is less than that of the latter group.

Table S3

| **CAL.L** | | | | | |
| --- | --- | --- | --- | --- | --- |
| Sample 1-Sample 2 | Test Statistic | Std. Error | Std. Test Statistic | Sig. | Adj. Sig.^a^ |
| 1-2 | -3.375 | 7.397 | -.456 | .648 | 1.000 |
| 1-3 | -20.501 | 7.527 | -2.724 | .006 | .019 |
| 2-3 | -17.126 | 6.972 | -2.456 | .014 | .042 |

Pairwise Comparisons of BBB leakage in CAL.L between eLCs, aLCs and HCs. After P value correction, there was statistical difference between aLCs and HCs (P=0.019), aLCs and eLCs (P=0.042), but there was no statistical difference between eLCs and HCs.

*CAL.L =* *Calcarine fissure and surrounding cortex.*

Table S4

| **SOG.L** | | | | | |
| --- | --- | --- | --- | --- | --- |
|  | Test Statistic | Std. Error | Std. Test Statistic | Sig. | Adj. Sig.^a^ |
| 1-2 | -3.676 | 7.397 | -.497 | .619 | 1.000 |
| 1-3 | -17.784 | 7.527 | -2.363 | .018 | .054 |
| 2-3 | -14.109 | 6.972 | -2.024 | .043 | .129 |

Pairwise Comparisons of BBB leakage in **SOG.L** between eLCs, aLCs and HCs. After P value correction, there was no statistical difference between eLCs, aLCs and HCs (P＞0.05).

*SOG.L=* *left Superior occipital gyrus*

Table S5

| **SOG.R** | | | | | |
| --- | --- | --- | --- | --- | --- |
|  | Test Statistic | Std. Error | Std. Test Statistic | Sig. | Adj. Sig.^a^ |
| 1-2 | -11.870 | 7.397 | -1.605 | .109 | .326 |
| 1-3 | -19.765 | 7.527 | -2.626 | .009 | .026 |
| 2-3 | -7.895 | 6.972 | -1.132 | .257 | .772 |

Pairwise Comparisons of BBB leakage in **SOG. R** between eLCs, aLCs and HCs. After P value correction, there was statistical difference between aLCs and HCs (P=0.026), but there was no statistical difference between eLCs and HCs, eLCs and aLCs (P＞0.05).

*SOG.R=* *right superior occipital gyrus*

Table S6

| **MOG.R** | | | | | |
| --- | --- | --- | --- | --- | --- |
|  | Test Statistic | Std. Error | Std. Test Statistic | Sig. | Adj. Sig.^a^ |
| 1-2 | -14.022 | 7.397 | -1.896 | .058 | .174 |
| 1-3 | -22.614 | 7.527 | -3.004 | .003 | .008 |
| 2-3 | -8.592 | 6.972 | -1.232 | .218 | .653 |

Pairwise Comparisons of BBB leakage in **MOG.R** between eLCs, aLCs and HCs. After P value correction, there was statistical difference between aLCs and HCs (P=0.008), but there was no statistical difference between eLCs and HCs, eLCs and aLCs (P＞0.05).

*MOG.R=right* *Middle occipital gyrus*

Table S7

| **IOG.L** | | | | | |
| --- | --- | --- | --- | --- | --- |
|  | Test Statistic | Std. Error | Std. Test Statistic | Sig. | Adj. Sig.^a^ |
| 1-2 | -3.985 | 7.397 | -.539 | .590 | 1.000 |
| 1-3 | -18.047 | 7.527 | -2.398 | .017 | .050 |
| 2-3 | -14.062 | 6.972 | -2.017 | .044 | .131 |

Pairwise Comparisons of BBB leakage in **IOG.L** between eLCs, aLCs and HCs. After P value correction, there was statistical difference between aLCs and HCs (P=0.050), but there was no statistical difference between eLCs and HCs, eLCs and aLCs (P＞0.05).

*IOG.L=* *left Inferior occipital gyrus，*

Table S8

| **IOG.R** | | | | | |
| --- | --- | --- | --- | --- | --- |
|  | Test Statistic | Std. Error | Std. Test Statistic | Sig. | Adj. Sig.^a^ |
| 1-2 | .205 | 7.397 | .028 | .978 | 1.000 |
| 1-3 | -19.000 | 7.527 | -2.524 | .012 | .035 |
| 2-3 | -19.205 | 6.972 | -2.755 | .006 | .018 |

Pairwise Comparisons of BBB leakage in **IOG.R** between eLCs, aLCs and HCs. After P value correction, there was statistical difference between aLCs and HCs(P=0.035), eLCs and aLCs (P=0.018), but there was no statistical difference between eLCs and HCs (P＞0.05).

*IOG.R=right Inferior occipital gyrus*

Table S9

| **TPOsup.L** | | | | | |
| --- | --- | --- | --- | --- | --- |
|  | Test Statistic | Std. Error | Std. Test Statistic | Sig. | Adj. Sig.^a^ |
| 1-2 | -21.637 | 7.397 | -2.925 | .003 | .010 |
| 1-3 | -14.066 | 7.527 | -1.869 | .062 | .185 |
| 2-3 | 7.571 | 6.972 | 1.086 | .278 | .833 |

Pairwise Comparisons of BBB leakage in **TPOsup.L** between eLCs, aLCs and HCs. After P value correction, there was statistical difference between eLCs and HCs(P=0.010), but there was no statistical difference between eLCs and HCs, aLCs and HCs (P＞0.05).

*TPOsup.L=* *left Temporal pole: superior temporal gyrus*

Table S10

| **TPOsup.R** | | | | | |
| --- | --- | --- | --- | --- | --- |
|  | Test Statistic | Std. Error | Std. Test Statistic | Sig. | Adj. Sig.^a^ |
| 1-2 | -16.462 | 7.527 | -2.187 | .029 | .086 |
| 1-3 | -16.851 | 7.397 | -2.278 | .023 | .068 |
| 2-3 | .389 | 6.972 | .056 | .956 | 1.000 |

Pairwise Comparisons of BBB leakage in **TPOsup.R** between eLCs, aLCs and HCs. After P value correction, there was no statistical difference between eLCs, aLCs and HCs (P＞0.05).

*TPOsup.R=right Temporal pole: superior temporal gyrus*

Table S11

| **TPOmid.L** | | | | | |
| --- | --- | --- | --- | --- | --- |
|  | Test Statistic | Std. Error | Std. Test Statistic | Sig. | Adj. Sig.^a^ |
| 1-2 | -18.379 | 7.397 | -2.485 | .013 | .039 |
| 1-3 | -14.407 | 7.527 | -1.914 | .056 | .167 |
| 2-3 | 3.972 | 6.972 | .570 | .569 | 1.000 |

Pairwise Comparisons of BBB leakage in **TPOmid.L** between eLCs, aLCs and HCs. After P value correction, there was statistical difference between eLCs and HCs(P=0.039), but there was no statistical difference between eLCs and HCs, aLCs and HCs (P＞0.05).

*TPOmid.L=* left *Temporal pole: middle temporal gyrus，*

Table S12

| **TPOmid.R** | | | | | |
| --- | --- | --- | --- | --- | --- |
|  | Test Statistic | Std. Error | Std. Test Statistic | Sig. | Adj. Sig.^a^ |
| 1-2 | -20.713 | 7.397 | -2.800 | .005 | .015 |
| 1-3 | -14.768 | 7.527 | -1.962 | .050 | .149 |
| 2-3 | 5.944 | 6.972 | .853 | .394 | 1.000 |

Pairwise Comparisons of BBB leakage in **TPOmid.L** between eLCs, aLCs and HCs. After P value correction, there was statistical difference between eLCs and HCs(P=0.015), but there was no statistical difference between eLCs and HCs, aLCs and HCs (P＞0.05).

*TPOmid.R=* right *Temporal pole: middle temporal gyrus，*

Table S13

| **Cerebelum_Crus1_L** | | | | | |
| --- | --- | --- | --- | --- | --- |
|  | Test Statistic | Std. Error | Std. Test Statistic | Sig. | Adj. Sig.^a^ |
| 1-2 | 2.214 | 7.397 | .299 | .765 | 1.000 |
| 1-3 | -20.402 | 6.972 | -2.926 | .003 | .010 |
| 2-3 | -18.188 | 7.527 | -2.416 | .016 | .047 |

Pairwise Comparisons of BBB leakage in **Cerebelum_Crus1_L** between eLCs, aLCs and HCs. After P value correction, there was statistical difference between aLCs and HCs(P=0.010), eLCs and aLCs (P=0.047), but there was no statistical difference between eLCs and HCs (P＞0.05).

Table S14

| **Cerebelum_Crus2_L** | | | | | |
| --- | --- | --- | --- | --- | --- |
|  | Test Statistic | Std. Error | Std. Test Statistic | Sig. | Adj. Sig.^a^ |
| 2-1 | 6.368 | 7.397 | .861 | .389 | 1.000 |
| 2-3 | -17.500 | 6.972 | -2.510 | .012 | .036 |
| 1-3 | -11.132 | 7.527 | -1.479 | .139 | .417 |

Pairwise Comparisons of BBB leakage in **Cerebelum_Crus2_L** between eLCs, aLCs and HCs. After P value correction, there was statistical difference between eLCs and aLCs (P=0.036), but there was no statistical difference between eLCs and HCs, aLCs and HCs (P＞0.05).

Table S15

| **Cerebelum_6_R** | | | | | |
| --- | --- | --- | --- | --- | --- |
|  | Test Statistic | Std. Error | Std. Test Statistic | Sig. | Adj. Sig.^a^ |
| 1-2 | -1.397 | 7.397 | -.189 | .850 | 1.000 |
| 1-3 | -18.559 | 7.527 | -2.466 | .014 | .041 |
| 2-3 | -17.162 | 6.972 | -2.462 | .014 | .042 |

Pairwise Comparisons of BBB leakage in **Cerebelum_Crus1_L** between eLCs, aLCs and HCs. After P value correction, there was statistical difference between aLCs and HCs(P=0.041), eLCs and aLCs (P=0.042), but there was no statistical difference between eLCs and HCs (P＞0.05).

Table S16

| **ktransGray** | | | | | |
| --- | --- | --- | --- | --- | --- |
|  | Test Statistic | Std. Error | Std. Test Statistic | Sig. | Adj. Sig.^a^ |
| 1-2 | -5.789 | 7.397 | -.783 | .434 | 1.000 |
| 1-3 | -21.970 | 7.527 | -2.919 | .004 | .011 |
| 2-3 | -16.182 | 6.972 | -2.321 | .020 | .061 |

Pairwise Comparisons of BBB leakage in **IOG.L** between eLCs, aLCs and HCs. After P value correction, there was statistical difference between aLCs and HCs (P=0.050), but there was no statistical difference between eLCs and HCs, eLCs and aLCs (P＞0.05).

*ktransGray= K^trans^ of gray.*

Table S17 Comparisons of cerebral cortex thickness between LCs and HCs

|  | HCs(N=29) | LCs(N=75) | t | P |
| --- | --- | --- | --- | --- |
| lh_G_and_S_cingul-Mid-Ant | 2.567±0.132 | 2.559±0.131 | 2.089 | 0.039 |
| lh_S_interm_prim-Jensen | 2.284±0.225 | 2.095±0.208 | 4.066 | 0.000 |
| lh_S_orbital_med-olfact_thickness | 2.277±0.162 | 2.209±0.118 | 2.319 | 0.022 |
| rh_G_postcentral_thickness | 2.175±0.128 | 2.113±0.138 | 2.037 | 0.041 |

Data are expressed as Mean±SD. The P values are obtained by using Independent Samples Test.

Comparisons of cerebral cortex thickness between LCs and HCs. There were significant differences in cortex thickness of 4 brain regions (P＜0.05).

*G_and_S_cingul-Mid-Ant= Middle-anterior part of the cingulate gyrus and sulcus (aMCC); S_interm_prim-Jensen=* *Sulcus intermedius primus (of Jensen);* lh_S_orbital_med-olfact= *left medial orbital sulcus (olfactory sulcus);* *rh_G_postcentral= right postcentral gyrus*

Table S18 Comparisons of cerebral cortex thickness between patients with LC at different stages and HCs

|  | HCs(N=29) | eLCs(N=39) | aLCs(N=36) | F | P |
| --- | --- | --- | --- | --- | --- |
| lh_G_and_S_transv_frontopol | 2.606±0.185 | 2.650±0.212 | 2.536±0.170 | 3.389 | 0.038 |
| lh_G_and_S_cingul-Mid-Ant | 2.567±0.132 | 2.530±0.139 | 2.483±0.120 | 3.448 | 0.036 |
| lh_G_Ins_lg_and_S_cent_ins | 2.984±0.236 | 3.099±0.235 | 2.970±0.248 | 3.247 | 0.043 |
| lh_Lat_Fis-post | 2.285±0.147 | 2.314±0.122 | 2.232±0.137 | 3.518 | 0.033 |
| lh_S_circular_insula_ant | 2.499±0.205 | 2.560±0.126 | 2.461±0.175 | 3.312 | 0.040 |
| lh_S_interm_prim-Jensen | 2.284±0.225 | 2.123±0.224 | 2.065±0.187 | 8.990 | 0.000 |
| lh_S_oc_middle_and_Lunatus | 1.948±0.096 | 1.970±0.122 | 1.900±0.085 | 4.508 | 0.013 |
| lh_S_oc_temp_med_and_Lingual | 2.288±0.111 | 2.322±0.155 | 2.241±0.119 | 3.546 | 0.032 |
| rh_G_occipital_sup | 2.175±0.129 | 2.209±0.135 | 2.133±0.109 | 3.510 | 0.034 |
| rh_S_central | 2.029±0.116 | 2.069±0.079 | 2.019±0.081 | 3.194 | 0.045 |
| rh_S_oc-temp_med_and_Lingual | 2.329±0.129 | 2.368±0.130 | 2.294±0.126 | 3.125 | 0.048 |

Data are expressed as Mean±SD. The P values are obtained by using ANOVA Test.

Comparisons of cerebral cortex thickness between eLC, aLC and HCs. There were significant differences in cortex thickness of 11 brain regions (P＜0.05).

*G_and_S_transv_frontopol=* *Transverse frontopolar gyri and sulci，G_and_S_cingul-Mid-Ant= Middle-anterior part of the cingulate gyrus and sulcus (aMCC)，G_Ins_lg_and_S_cent_ins=* *Long insular gyrus and central sulcus of the insula，Lat_Fis-post=* *Posterior ramus (or segment) of the lateral sulcus (orfissure)，S_circular_insula_ant=* *Anterior segment of the circular sulcus of the insula，S_interm_prim-Jensen=* *Sulcus intermedius primus (of Jensen)，S_oc_middle_and_Lunatus=* *Middle occipital sulcus and lunatus sulcus，S_oc_temp_med_and_Lingual=* *Medial occipito-temporal sulcus (collateral sulcus) and lingual sulcus，G_occipital_sup=* *Superior occipital gyrus (O1)，S_central=* *Central sulcus (Rolando'sfissure)*

Table S19 Multiple comparisons of cerebral cortex thickness between patients with LC at different stages and HCs, The P values are obtained by using LSD Test. 1= HCs; 2= eLCs; 3= aLCs.

|  | group | Mean Difference | Std. Error | Sig. | 95% Confidence Interval | |
| --- | --- | --- | --- | --- | --- | --- |
|  |  |  |  |  | Lower Bound | Upper Bound |
| lh_G_and_S_transv_frontopol_thickness | 1vs2 | -0.044 | 0.047 | 0.349 | -0.137 | 0.049 |
|  | 1vs3 | 0.070 | 0.048 | 0.143 | -0.024 | 0.165 |
|  | 2vs3 | 0.114 | 0.044 | 0.011* | 0.027 | 0.202 |
| lh_G_and_S_cingul-Mid-Ant_thickness | 1vs2 | 0.037 | 0.032 | 0.246 | -0.026 | 0.101 |
|  | 1vs3 | 0.085 | 0.033 | 0.011* | 0.020 | 0.149 |
|  | 2vs3 | 0.047 | 0.030 | 0.119 | -0.012 | 0.107 |
| lh_G_Ins_lg_and_S_cent_ins_thickness | 1vs2 | -0.116 | 0.059 | 0.052 | -0.232 | 0.001 |
|  | 1vs3 | 0.014 | 0.060 | 0.820 | -0.105 | 0.132 |
|  | 2vs3 | 0.129 | 0.055 | 0.022* | 0.019 | 0.239 |
| lh_Lat_Fis-post_thickness | 1vs2 | -0.028 | 0.033 | 0.391 | -0.094 | 0.037 |
|  | 1vs3 | 0.053 | 0.033 | 0.116 | -0.013 | 0.120 |
|  | 2vs3 | 0.082 | 0.031 | 0.010* | 0.020 | 0.143 |
| lh_S_circular_insula_ant_thickness | 1vs2 | -0.060 | 0.041 | 0.147 | -0.142 | 0.021 |
|  | 1vs3 | 0.039 | 0.042 | 0.357 | -0.044 | 0.122 |
|  | 2vs3 | 0.099 | 0.039 | 0.012* | 0.022 | 0.176 |
| lh_S_interm_prim-Jensen_thickness | 1vs2 | 0.161 | 0.052 | 0.002* | 0.058 | 0.265 |
|  | 1vs3 | 0.219 | 0.053 | 0.000* | 0.114 | 0.324 |
|  | 2vs3 | 0.058 | 0.049 | 0.243 | -0.040 | 0.155 |
| lh_S_oc_middle_and_Lunatus_thickness | 1vs2 | -0.022 | 0.025 | 0.378 | -0.073 | 0.028 |
|  | 1vs3 | 0.048 | 0.026 | 0.064 | -0.003 | 0.099 |
|  | 2vs3 | 0.071 | 0.024 | 0.004* | 0.023 | 0.118 |
| lh_S_oc-temp_med_and_Lingual_thickness | 1vs2 | -0.034 | 0.032 | 0.293 | -0.098 | 0.030 |
|  | 1vs3 | 0.047 | 0.033 | 0.158 | -0.018 | 0.112 |
|  | 2vs3 | 0.081 | 0.030 | 0.009* | 0.021 | 0.141 |
| rh_G_occipital_sup_thickness | 1vs2 | -0.035 | 0.031 | 0.258 | -0.095 | 0.026 |
|  | 1vs3 | 0.042 | 0.031 | 0.185 | -0.020 | 0.103 |
|  | 2vs3 | 0.076 | 0.029 | 0.009* | 0.019 | 0.134 |
| rh_S_central_thickness | 1vs2 | -0.040 | 0.022 | 0.077 | -0.084 | 0.004 |
|  | 1vs3 | 0.011 | 0.023 | 0.639 | -0.034 | 0.056 |
|  | 2vs3 | 0.051 | 0.021 | 0.018* | 0.009 | 0.092 |
| rh_S_oc-temp_med_and_Lingual_thickness | 1vs2 | -0.039 | 0.031 | 0.222 | -0.101 | 0.024 |
|  | 1vs3 | 0.035 | 0.032 | 0.271 | -0.028 | 0.099 |
|  | 2vs3 | 0.074 | 0.030 | 0.014* | 0.015 | 0.133 |

Multiple comparisons of cerebral cortex thickness between eLCs, aLCs and HCs. Compared with HCs, the cortical thickness of the left long insular gyrus and the central sulcus of the insula increased in eLC group (P<0.05). The cortex thickness of left aMCC and the left sulcus intermedius primus in aLC group decreased (P<0.05). Compared with eLC group, The cortical thickness of 9 brain regions decreased in aLC group (*i.e., left transverse frontopolar gyri and sulci, left Long insular gyrus and central sulcus of the insula, left Posterior ramus of the lateral sulcus, left Anterior segment of the circular sulcus of the insula, left Middle occipital sulcus and lunatus sulcus, left Medial occipito-temporal sulcus and lingual sulcus, right Superior occipital gyrus, right Central sulcus and right Medial occipito-temporal sulcus and lingual sulcus)(P<0.05*).

*G_and_S_transv_frontopol=* *Transverse frontopolar gyri and sulci，G_and_S_cingul-Mid-Ant= Middle-anterior part of the cingulate gyrus and sulcus (aMCC)，G_Ins_lg_and_S_cent_ins=* *Long insular gyrus and central sulcus of the insula，Lat_Fis-post=* *Posterior ramus (or segment) of the lateral sulcus (orfissure)，S_circular_insula_ant=* *Anterior segment of the circular sulcus of the insula，S_interm_prim-Jensen=* *Sulcus intermedius primus (of Jensen)，S_oc_middle_and_Lunatus=* *Middle occipital sulcus and lunatus sulcus，S_oc_temp_med_and_Lingual=* *Medial occipito-temporal sulcus (collateral sulcus) and lingual sulcus，G_occipital_sup=* *Superior occipital gyrus (O1)，S_central=* *Central sulcus (Rolando'sfissure)*

Table S20 Comparison of subcortical structure volume between LCs and HCs

|  | HCs(N=29) | LCs(N=75) | t | P |
| --- | --- | --- | --- | --- |
| Left-Inf-Lat-Vent | 0.023±0.008 | 0.029±0.018 | -2.609 | 0.010 |
| Left-Thalamus | 0.486±0.045 | 0.456±0.045 | 3.176 | 0.002 |
| Left-Pallidum | 0.124±0.013 | 0.115±0.013 | 2.734 | 0.007 |
| Left-Hippocampus | 0.276±0.024 | 0.261±0.030 | 2.244 | 0.027 |
| Left-VentralDC | 0.279±0.021 | 0.261±0.023 | 3.840 | 0.000 |
| Right-Cerebellum-White-Matter | 0.849±0.089 | 0.793±0.010 | 2.614 | 0.010 |
| Right-Thalamus | 0.461±0.039 | 0.439±0.042 | 2.455 | 0.016 |
| Right-Putamen | 0.331±0.035 | 0.311±0.041 | 2.319 | 0.022 |
| Right-Pallidum | 0.122±0.013 | 0.113±0.015 | 2.928 | 0.004 |
| Right-Hippocampus | 0.288±0.028 | 0.275±0.029 | 2.053 | 0.043 |
| Right-Accumbens-area | 0.038±0.006 | 0.034±0.006 | 2.817 | 0.006 |
| Right-VentralDC | 0.279±0.023 | 0.263±0.022 | 2.901 | 0.004 |

Data are expressed as Mean±SD. The P values are obtained by using Independent Samples Test.

Comparisons of volume of subcortical structure between LCs and HCs. There were significant differences in 12 brain regions (P＜0.05).


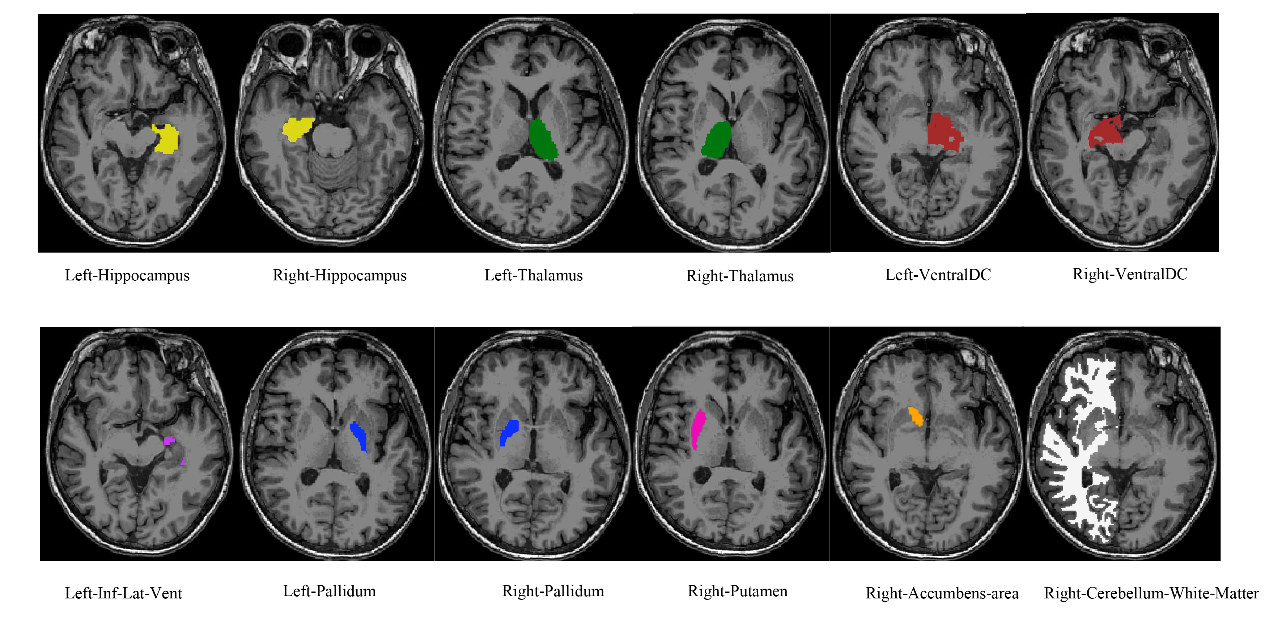


Figure S1 Comparisons of volume of subcortical structure between LCs and HCs

Table S21 Comparison of subcortical structure volume between patients with LC at different stages and HCs

|  | HCs(N=29) | eLCs(N=39) | aLCs(N=36) | F | P |
| --- | --- | --- | --- | --- | --- |
| Left-Inf-Lat-Vent | 0.023±0.008 | 0.026±0.012 | 0.034±0.023 | 4.45 | 0.014 |
| Left-Thalamus | 0.486±0.045 | 0.468±0.039 | 0.443±0.041 | 8.743 | 0.000 |
| Left-Putamen | 0.327±0.038 | 0.319±0.031 | 0.303±0.041 | 3.583 | 0.031 |
| Left-Pallidum | 0.124±0.013 | 0.119±0.013 | 0.112±0.013 | 6.639 | 0.002 |
| Left-Hippocampus | 0.276±0.024 | 0.267±0.030 | 0.256±0.031 | 3.768 | 0.026 |
| Left-Amygdala | 0.111±0.011 | 0.109±0.012 | 0.104±0.013 | 3.337 | 0.040 |
| Left-VentralDC | 0.279±0.021 | 0.264±0.020 | 0.256±0.025 | 8.661 | 0.000 |
| Right-Cerebellum-White-Matter | 0.849±0.089 | 0.809±0.092 | 0.775±0.111 | 4.543 | 0.013 |
| Right-Thalamus | 0.461±0.039 | 0.450±0.037 | 0.428±0.044 | 5.823 | 0.004 |
| Right-Putamen | 0.331±0.035 | 0.318±0.035 | 0.304±0.047 | 3.904 | 0.023 |
| Right-Pallidum | 0.122±0.013 | 0.117±0.013 | 0.108±0.015 | 8.482 | 0.000 |
| Right-Accumbens-area | 0.038±0.006 | 0.035±0.005 | 0.033±0.007 | 5.796 | 0.004 |
| Right-VentralDC | 0.279±0.023 | 0.267±0.021 | 0.260±0.024 | 5.081 | 0.008 |
| Right-choroid-plexus | 0.040±0.010 | 0.040±0.012 | 0.049±0.016 | 5.458 | 0.006 |

Data are expressed as Mean±SD. The P values are obtained by using ANOVA Test.

Comparisons of volume of subcortical structure between eLC, aLC and HCs. There were significant differences in 14 brain regions (P＜0.05).

Table S22 Multiple comparisons of subcortical structure volume between patients with LC at different stages and HCs，The P values are obtained by using LSD Test. 1= HCs; 2= eLCs; 3= aLCs.

|  | group | Mean Difference | Std. Error | Sig. | 95% Confidence Interval | |
| --- | --- | --- | --- | --- | --- | --- |
|  |  |  |  |  | Lower Bound | Upper Bound |
| Left-Inf-Lat-Vent | 1vs2 | -2.69E-05 | 3.91E-05 | 0.493 | -1.04E-04 | 5.06E-05 |
|  | 1vs3 | -1.11E-04 | 3.98E-05 | 0.006* | -1.90E-04 | -3.19E-05 |
|  | 2vs3 | -8.38E-05 | 3.68E-05 | 0.025* | -1.57E-04 | -1.08E-05 |
| Left-Thalamus | 1vs2 | 1.75E-04 | 1.02E-04 | 0.089 | -2.70E-05 | 3.76E-04 |
|  | 1vs3 | 4.26E-04 | 1.03E-04 | 0.000* | 2.21E-04 | 6.31E-04 |
|  | 2vs3 | 2.51E-04 | 9.58E-05 | 0.010* | 6.12E-05 | 4.41E-04 |
| Left-Putamen | 1vs2 | 8.03E-05 | 8.89E-05 | 0.368 | -9.60E-05 | 2.57E-04 |
|  | 1vs3 | 2.34E-04 | 9.05E-05 | 0.011* | 5.49E-05 | 4.14E-04 |
|  | 2vs3 | 1.54E-04 | 8.38E-05 | 0.069 | -1.22E-05 | 3.20E-04 |
| Left-Pallidum | 1vs2 | 4.51E-05 | 3.15E-05 | 0.155 | -1.74E-05 | 1.07E-04 |
|  | 1vs3 | 1.14E-04 | 3.20E-05 | 0.001* | 5.09E-05 | 1.78E-04 |
|  | 2vs3 | 6.94E-05 | 2.97E-05 | 0.021* | 1.06E-05 | 1.28E-04 |
| Left-Hippocampus | 1vs2 | 9.15E-05 | 6.98E-05 | 0.193 | -4.70E-05 | 2.30E-04 |
|  | 1vs3 | 1.94E-04 | 7.11E-05 | 0.007* | 5.31E-05 | 3.35E-04 |
|  | 2vs3 | 1.03E-04 | 6.58E-05 | 0.122 | -2.80E-05 | 2.33E-04 |
| Left-Amygdala | 1vs2 | 1.95E-05 | 2.88E-05 | 0.501 | -3.77E-05 | 7.66E-05 |
|  | 1vs3 | 7.16E-05 | 2.93E-05 | 0.016* | 1.34E-05 | 1.30E-04 |
|  | 2vs3 | 5.21E-05 | 2.72E-05 | 0.058 | -1.78E-06 | 1.06E-04 |
| Left-VentralDC | 1vs2 | 1.50E-04 | 5.46E-05 | 0.007* | 4.18E-05 | 2.58E-04 |
|  | 1vs3 | 2.30E-04 | 5.56E-05 | 0.000* | 1.19E-04 | 3.40E-04 |
|  | 2vs3 | -2.30E-04 | 5.56E-05 | 0.000* | -3.40E-04 | -1.19E-04 |
| Right-Cerebellum-White-Matter | 1vs2 | 4.04E-04 | 2.41E-04 | 0.096 | -7.37E-05 | 8.82E-04 |
|  | 1vs3 | 7.39E-04 | 2.45E-04 | 0.003* | 2.53E-04 | 1.23E-03 |
|  | 2vs3 | 3.35E-04 | 2.27E-04 | 0.144 | -1.16E-04 | 7.85E-04 |
| Right-Thalamus | 1vs2 | 1.17E-04 | 9.84E-05 | 0.237 | -7.82E-05 | 3.12E-04 |
|  | 1vs3 | 3.32E-04 | 1.00E-04 | 0.001* | 1.33E-04 | 5.30E-04 |
|  | 2vs3 | 2.15E-04 | 9.27E-05 | 0.023* | 3.07E-05 | 3.99E-04 |
| Right-Putamen | 1vs2 | 1.34E-04 | 9.67E-05 | 0.169 | -5.77E-05 | 3.26E-04 |
|  | 1vs3 | 2.74E-04 | 9.84E-05 | 0.006* | 7.89E-05 | 4.69E-04 |
|  | 2vs3 | 1.40E-04 | 9.12E-05 | 0.128 | -4.08E-05 | 3.21E-04 |
| Right-Pallidum | 1vs2 | 4.84E-05 | 3.38E-05 | 0.155 | -1.87E-05 | 1.15E-04 |
|  | 1vs3 | 1.37E-04 | 3.44E-05 | 0.000* | 6.92E-05 | 2.06E-04 |
|  | 2vs3 | 8.90E-05 | 3.18E-05 | 0.006* | 2.59E-05 | 1.52E-04 |
| Right-Accumbens-area | 1vs2 | 2.51E-05 | 1.48E-05 | 0.092 | -4.20E-06 | 5.45E-05 |
|  | 1vs3 | 5.11E-05 | 1.51E-05 | 0.001* | 2.12E-05 | 8.10E-05 |
|  | 2vs3 | 2.60E-05 | 1.39E-05 | 0.066 | -1.70E-06 | 5.36E-05 |
| Right-VentralDC | 1vs2 | 1.11E-04 | 5.51E-05 | 0.047* | 1.75E-06 | 2.20E-04 |
|  | 1vs3 | 1.78E-04 | 5.61E-05 | 0.002* | 6.69E-05 | 2.89E-04 |
|  | 2vs3 | 6.71E-05 | 5.20E-05 | 0.200 | -3.60E-05 | 1.70E-04 |
| Right-choroid-plexus | 1vs2 | 2.30E-06 | 3.18E-05 | 0.942 | -6.07E-05 | 6.53E-05 |
|  | 1vs3 | -8.69E-05 | 3.23E-05 | 0.008* | -1.51E-04 | -2.28E-05 |
|  | 2vs3 | -8.92E-05 | 2.99E-05 | 0.004* | -1.49E-04 | -2.98E-05 |

Multiple comparisons of volume of subcortical structure between eLCs, aLCs and HCs. Compared with HCs, the volume of 12 brain regions in aLC group decreased (*i.e., bilateral thalamus, putamen, pallidum, ventral diencephalon, left hippocampus, amygdala and right cerebellum white matter, nucleus accumbens*), whereas the volume of *the left temporal horn of lateral ventricle* increased (P<0.05); the volume of the bilateral Ventral Diencephalon decreased in eLC group (P<0.05). Compared with eLC group, the volumes of the *bilateral thalamus, right pallidum, and right choroid plexus* in aLC group (P<0.05).


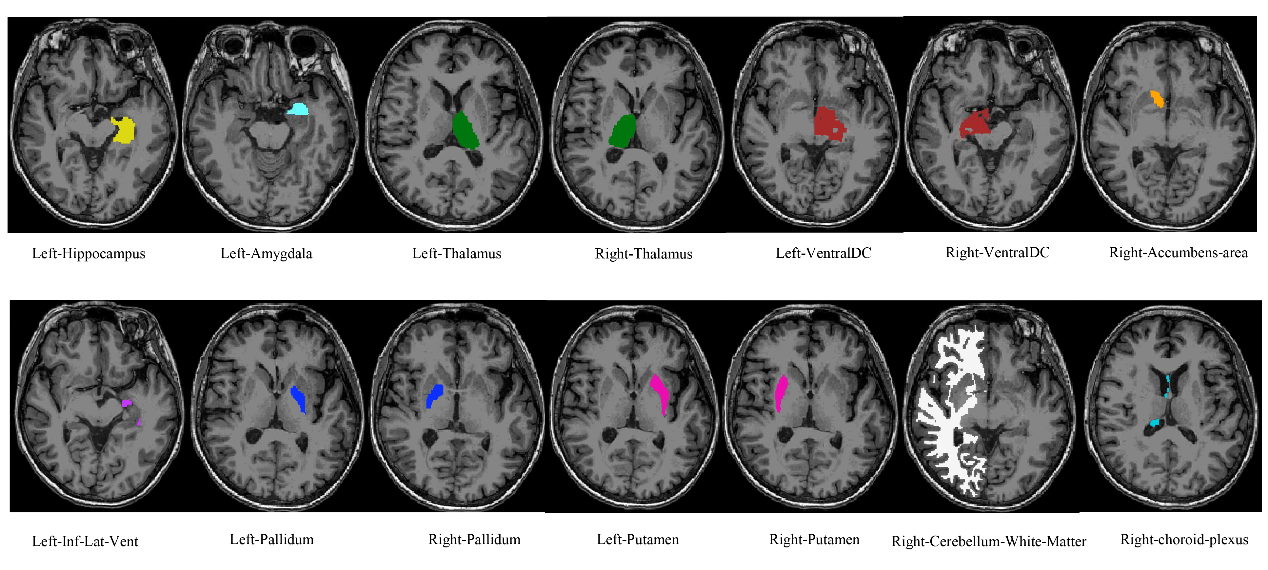


Figure S2 Comparisons of volume of subcortical structure between aLCs and HCs


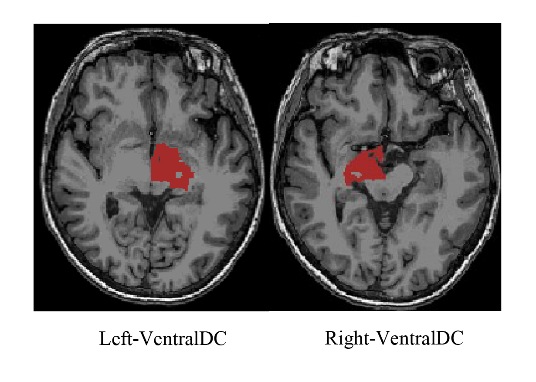


Figure S3 Comparisons of volume of subcortical structure between eLCs and HCs


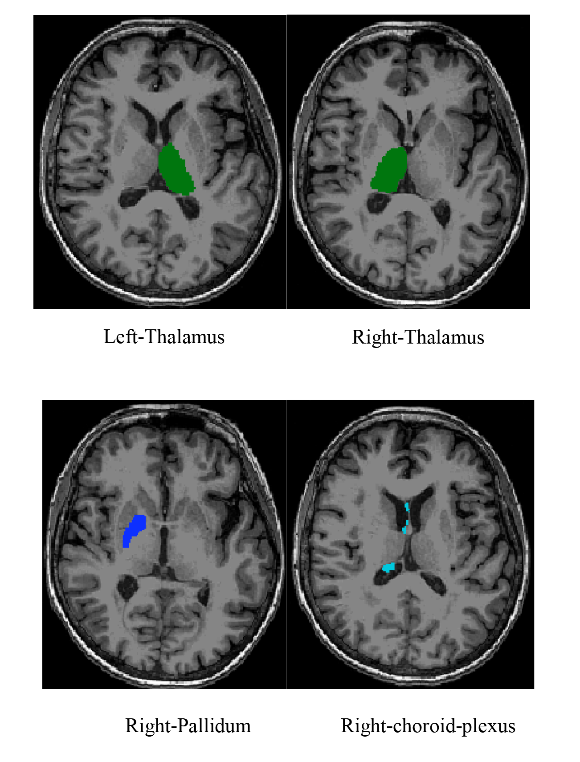


Figure S4 Comparisons of volume of subcortical structure between eLCs and aLCs

Table S23. Correlation between differential cerebral cortical thickness, subcortical volume and BBB leakage

| BBB | Cortical thickness and volume of subcortical structure | R | P |
| --- | --- | --- | --- |
| Calcarine_L | lh_Lat_Fis-post_thickness  rh_S_central_thickness  Right-Thalamus  lh_Lat_Fis-post_thickness  Left-Inf-Lat-Vent  Left-Thalamus  Right-Thalamus  Right-Thalamus  Left-Inf-Lat-Vent  Left-Thalamus  Left-VentralDC  Right-Thalamus  Right-VentralDC  lh_Lat_Fis-post_thickness  Left-Thalamus  Left-VentralDC  Right-Cerebellum-White-Matter  Right-Thalamus  Left-Inf-Lat-Vent  Left-Thalamus  Right-Thalamus  lh_G_and_S_cingul-Mid-Ant_thickness  Left-Thalamus  Left-VentralDC  Right-Thalamus  lh_G_and_S_cingul-Mid-Ant_thickness  lh_Lat_Fis-post_thickness  lh_S_circular_insula_ant_thickness  rh_G_occipital_sup_thickness  Left-Inf-Lat-Vent  Left-Thalamus  Left-Hippocampus  Left-Amygdala  Left-VentralDC  Right-Cerebellum-White-Matter  Right-Thalamus  Right-VentralDC  lh_G_and_S_cingul-Mid-Ant_thickness  Left-Amygdala  Left-VentralDC  Right-Thalamus  lh_Lat_Fis-post_thickness  Left-Inf-Lat-Vent  Left-Thalamus  Left-Amygdala  Left-VentralDC  Right-Thalamus  Right-VentralDC  lh_G_and_S_transv_frontopol_thickness  lh_G_and_S_cingul-Mid-Ant_thickness  lh_Lat_Fis-post_thickness  lh_S_interm_prim-Jensen_thickness  lh_S_oc-temp_med_and_Lingual_thickness  Left-Thalamus  Left-Hippocampus  Left-VentralDC  Right-Cerebellum-White-Matter  Right-Thalamus  Right-Accumbens-area  Right-VentralDC  lh_G_and_S_cingul-Mid-Ant_thickness  Left-VentralDC  Right-Cerebellum-White-Matter  Right-Thalamus  Right-VentralDC  lh_G_and_S_cingul-Mid-Ant_thickness  lh_G_Ins_lg_and_S_cent_ins_thickness  lh_Lat_Fis-post_thickness  lh_S_interm_prim-Jensen_thickness  lh_S_oc-temp_med_and_Lingual_thickness  rh_G_occipital_sup_thickness  Left-Inf-Lat-Vent  Left-Thalamus  Left-Pallidum  Left-Hippocampus  Left-VentralDC  Right-Cerebellum-White-Matter  Right-Thalamus  Right-Accumbens-area  Right-VentralDC  lh_G_and_S_cingul-Mid-Ant_thickness  Left-Thalamus  Right-Thalamus | -0.336 | 0.001 |
|  |  | -0.239 | 0.015 |
|  |  | -0.212 | 0.032 |
| Occipital_Sup_L |  | -0.261 | 0.008 |
|  |  | 0.270 | 0.006 |
|  |  | -0.275 | 0.005 |
|  |  | -0.284 | 0.004 |
| Occipital_Sup_R |  | -0.205 | 0.038 |
| Occipital_Mid_R |  | 0.281 | 0.004 |
|  |  | -0.231 | 0.019 |
|  |  | -0.266 | 0.007 |
|  |  | -0.346 | 0.000 |
|  |  | -0.232 | 0.018 |
| Occipital_Inf_L |  | -0.239 | 0.015 |
|  |  | -0.355 | 0.000 |
|  |  | -0.233 | 0.018 |
|  |  | -0.220 | 0.025 |
|  |  | -0.313 | 0.001 |
| Occipital_Inf_R |  | 0.324 | 0.001 |
|  |  | -0.216 | 0.029 |
|  |  | -0.200 | 0.043 |
| Temporal_Pole_Sup_L |  | -0.213 | 0.031 |
|  |  | -0.211 | 0.033 |
|  |  | -0.250 | 0.011 |
|  |  | -0.249 | 0.011 |
| Temporal_Pole_Sup_R |  | -0.230 | 0.019 |
|  |  | -0.271 | 0.006 |
|  |  | -0.207 | 0.036 |
|  |  | -0.202 | 0.010 |
|  |  | 0.274 | 0.005 |
|  |  | -0.332 | 0.001 |
|  |  | -0.275 | 0.005 |
|  |  | -0.305 | 0.002 |
|  |  | -0.341 | 0.000 |
|  |  | -0.262 | 0.007 |
|  |  | -0.340 | 0.000 |
|  |  | -0.303 | 0.002 |
| Temporal_Pole_Mid_L |  | -0.234 | 0.018 |
|  |  | -0.232 | 0.018 |
|  |  | -0.259 | 0.008 |
|  |  | -0.229 | 0.020 |
| Temporal_Pole_Mid_R |  | -0.240 | 0.015 |
|  |  | 0.257 | 0.009 |
|  |  | -0.309 | 0.002 |
|  |  | -0.288 | 0.003 |
|  |  | -0.291 | 0.003 |
|  |  | -0.332 | 0.001 |
|  |  | -0.250 | 0.011 |
| Cerebelum_Crus1_L |  | -0.234 | 0.017 |
|  |  | -0.384 | 0.000 |
|  |  | -0.254 | 0.010 |
|  |  | -0.227 | 0.021 |
|  |  | -0.264 | 0.007 |
|  |  | -0.352 | 0.000 |
|  |  | -0.256 | 0.009 |
|  |  | -0.325 | 0.001 |
|  |  | -0.304 | 0.002 |
|  |  | -0.433 | 0.000 |
|  |  | -0.265 | 0.007 |
|  |  | -0.282 | 0.004 |
| Cerebelum_Crus2_L |  | -0.213 | 0.031 |
|  |  | -0.281 | 0.004 |
|  |  | -0.272 | 0.005 |
|  |  | -0.244 | 0.013 |
|  |  | -0.229 | 0.020 |
| Cerebelum_6_R |  | -0.375 | 0.000 |
|  |  | -0.206 | 0.037 |
|  |  | -0.231 | 0.019 |
|  |  | -0.265 | 0.007 |
|  |  | -0.301 | 0.002 |
|  |  | -0.271 | 0.006 |
|  |  | 0.218 | 0.027 |
|  |  | -0.402 | 0.000 |
|  |  | -0.227 | 0.021 |
|  |  | -0.226 | 0.022 |
|  |  | -0.291 | 0.003 |
|  |  | -0.291 | 0.003 |
|  |  | -0.434 | 0.000 |
|  |  | -0.276 | 0.005 |
|  |  | -0.238 | 0.016 |
| ktransGray |  | -0.208 | 0.035 |
|  |  | -0.205 | 0.038 |
|  |  | -0.274 | 0.005 |

The P values are obtained by using spearman correlation analysis, The correlation is significant at the 0.05 level.

The brain regions with increased BBB permeability were negatively correlated with the cortex thickness and the volume of subcortical structure (P<0.05).

Table S24.Correlation between tumor markers, diameter and BBB leakage in patients with LC

| BBB | tumor markers and diameter | R | P |
| --- | --- | --- | --- |
| Calcarine_L | CYFRA21-1 | 0.266 | 0.021 |
| Occipital_Sup_L |  | 0.235 | 0.042 |
| Temporal_Pole_Mid_L |  | 0.449 | 0.000 |
| Temporal_Pole_Mid_R |  | 0.508 | 0.000 |
| Cerebelum_Crus1_L |  | 0.303 | 0.008 |
| Cerebelum_6_R |  | 0.342 | 0.003 |
| ktransGray |  | 0.305 | 0.008 |
| ktransGray | CEA | 0.228 | 0.049 |
| Calcarine_L | Tumor diameter | 0.257 | 0.026 |
| Occipital_Inf_L |  | 0.239 | 0.039 |
| Occipital_Inf_R |  | 0.298 | 0.009 |
| Temporal_Pole_Mid_L |  | 0.599 | 0.000 |
| Temporal_Pole_Mid_R |  | 0.659 | 0.000 |
| Cerebelum_Crus1_L |  | 0.329 | 0.004 |
| Cerebelum_Crus2_L |  | 0.299 | 0.009 |

The P values are obtained by using spearman correlation analysis, The correlation is significant at the 0.05 level.

The maximum diameter of the tumor was positively correlated with K^trans^ (CAL.L, IOG.L, IOG.R, TPOmid.L, left Cerebellum Crus2). CEA was positively correlated with K^trans^ of the cerebral gray matter, and CYFRA21-1 was positively correlated with CAL.L, TPOmid.L, left cerebellum crus1, right cerebellum 6, and Ktrans Gray (P<0.05, R=0.25–0.51)
